# Supplementary material for: Disorder enhances the fracture toughness of 2D mechanical metamaterials
Source: PNAS Nexus. 2025 Jan 28;4(2):pgaf023. doi: 10.1093/pnasnexus/pgaf023 (PMC11803419; doi:10.1093/pnasnexus/pgaf023)
Supplement: pgaf023_Supplementary_Data [file pgaf023_supplementary_data.zip › PNASNEXUS-PNASNEXUS-2024-01136R-s05.pdf]

PNAS nexus

Supplementary Information for

Disorder Enhances the Fracture Toughness of Two-Dimensional Mechanical Metamaterials

Sage Fulco, Michal K. Budzik, Hongyi Xiao, Douglas J. Durian, and Kevin T. Turner

Sage Fulco & Kevin T. Turner.
E-mails: fulco@seas.upenn.edu (S. Fulco) & kturner@seas.upenn.edu (K.T. Turner)

This PDF file includes:

Supplementary text
Figs. S1 to S6
Legends for Movies S1 to S4

Other supplementary materials for this manuscript include the following:

Movies S1 to S4

Supporting Information Text

1. Specimen Fabrication

As described in the Materials and Methods section, experimental specimens were laser-cut from from 6 mm-thick, cast, transparent polymethylmethacrylate (PMMA) sheets. The cut ligaments were measured optically to have a thickness of $t = 0.55$ mm $\pm$ 0.05 mm and a length of $L = 4.95$ mm $\pm$ 0.05, as shown in Fig. S1. This closely matches the nominal dimensions used in the finite element simulations of $t = 0.5$ mm, and $L = 5$ mm.

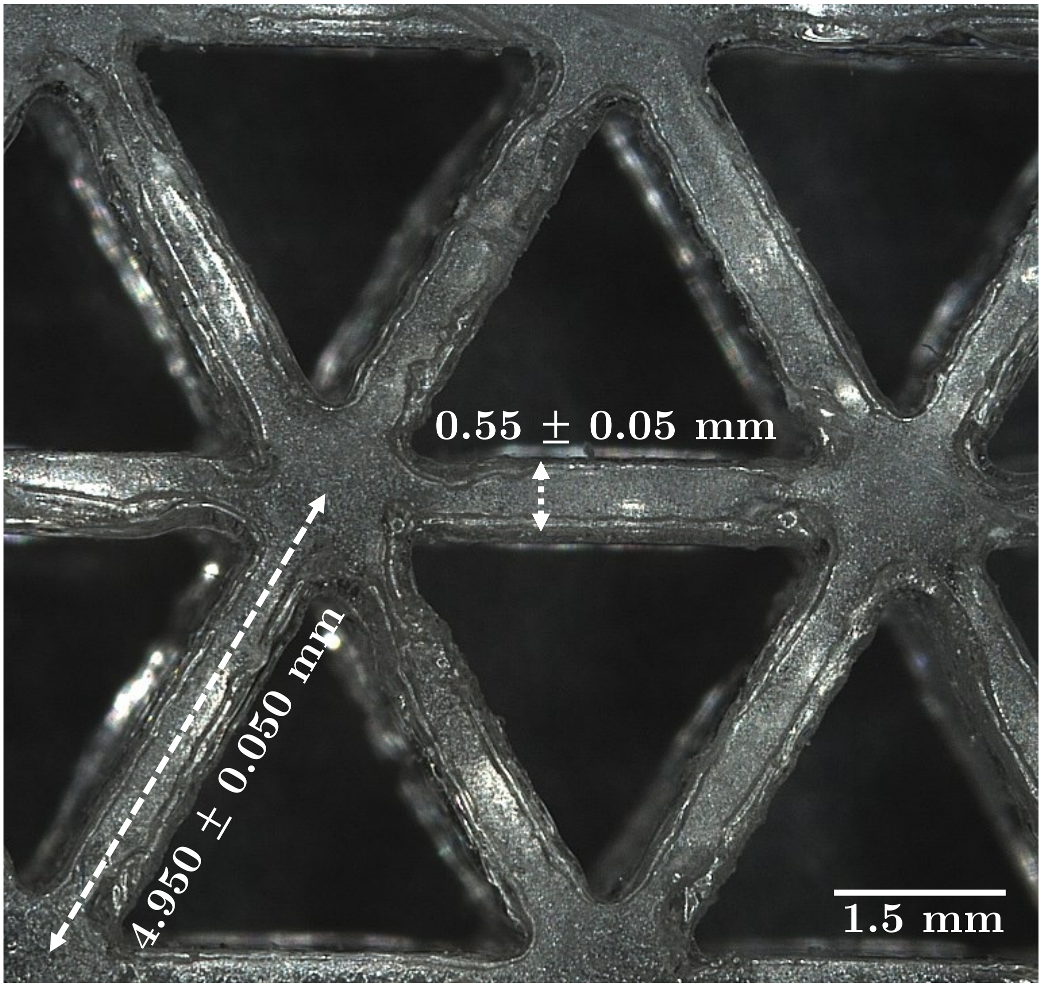

Fig. S1. Optical image of laser-cut PMMA lattice (without raster cuts) with ligament width and unit cell length indicated.

2. Model Generation and Finite Element Simulations

Disordered lattices were generated from the ordered lattice by introducing a disorder “seed”, as described in the Materials and Methods section and shown in Fig. S2. The seed describes the direction and relative magnitudes of the node perturbations. To generate lattices of varying disorder, the average node perturbation in the seed is scaled and applied to the ordered lattice. By considering a large number of seed geometries scaled to varying levels of disorder, as was done in the finite element simulations (∼60 seeds at 7 levels of disorder for a total of 420 unique geometries) allows for a consideration of the effect of disorder across a variety of geometries. Comparing specimens from the same seeds at different levels of disorder, as was done in the experiments, elucidates solely the effects arising from the magnitude of disorder, without introducing any bias that might arise from a changing geometry.

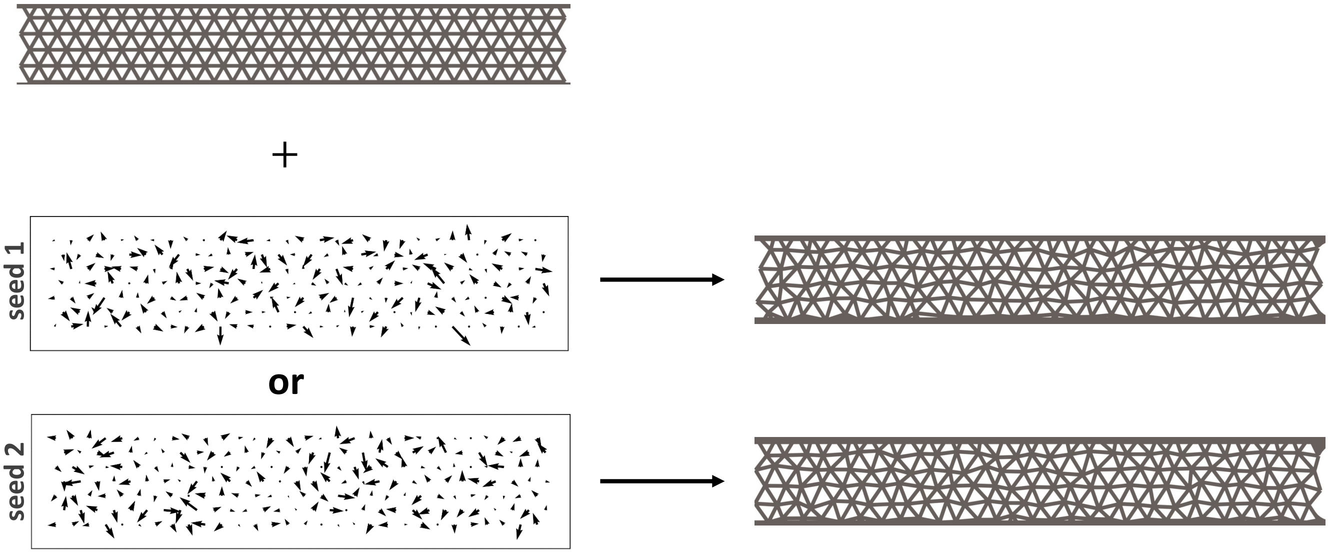

Fig. S2. Representative disordered lattices used for the experiments, with $\bar{\delta} \approx 0.15$ in both cases. To generate the disordered lattices, an ordered lattice is perturbed following the directions and relative magnitudes shown for each seed. To generate lattices of varying levels of disorder, the average magnitude of the perturbations is changed.

The overall specimen used for simulations and experiments consisted of a lattice with 30 horizontal nodes and 5 vertical nodes, with a unit cell size of $L = 5$ mm and ligament thickness $t = 0.5$ mm. The lattice is confined between two solid beams, each 13 mm thick and 225 mm long, with loading points 175 mm from the far edge of the lattice. A representative finite element geometry with an ordered lattice is shown in the Fig. S3, with the boundary conditions indicated. The bottom mounting hole center is fixed in x and y, while the top mounting hole center has a prescribed displacement of 0 in x and 1 mm in y. The ligament stresses and associated forces and displacements are scaled using linearity to predict failure, as described in the Materials and Methods section. The surfaces of the mounting holes are constrained to the hole centers using a coupling constraint, simulating a pin-mounting, as was used in the fracture experiments.

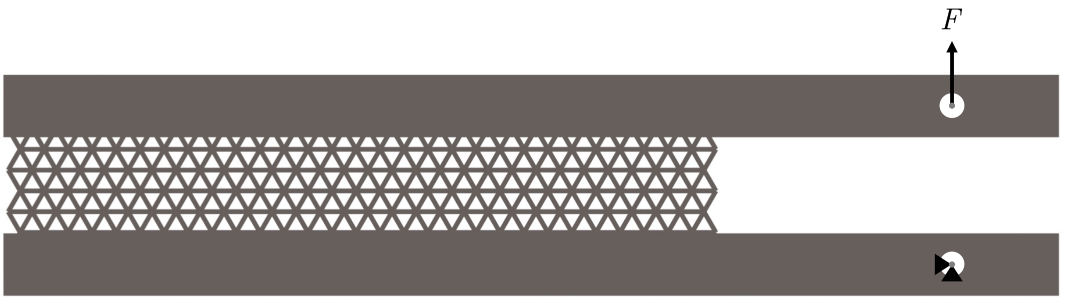

Fig. S3. Double cantilever beam specimen geometry used for simulations and experiments, with an ordered lattice. The lower loading point is fixed horizontally and vertically, and the top loading point is displaced vertically, causing a vertical reaction force, F.

3. Unit Cell Failure Analysis

The analytical model described in the section "Effect of Disorder on Local Lattice Failure" predicts the failure strength of triangular unit cells, with perturbations of varying magnitude. These are verified through finite element simulations, as shown in Fig. 2, where the average and standard deviations in strength are given as a function of the magnitude of the perturbation. While the average strength is the most pertinent result for a large lattice with perturbations in random directions, the analytical model is capable of predicting the strength as a function of perturbations at specific angles.

Figure S5 shows the complete set of finite element simulation results, showing the strength of the perturbed unit cell, relative to an ordered unit cell, for perturbations of varying magnitudes and directions. The analytical model predictions of the strength as a function of perturbation magnitude for each specific angle are indicated by the solid lines on the figure. The analytical and finite element results are of similar magnitude and trend, with larger perturbations resulting in lower strength. Notably, the analytical model and finite element results differ most significantly for small perturbations at angles between 30-60º. This is a result of the greater variation in stresses within ligaments for perturbations in these directions due to stress concentrations at the edges of the ligaments, which is not captured by the analytical model. This results in the analytical model slightly over-predicting the strength of the unit cell in these cases.

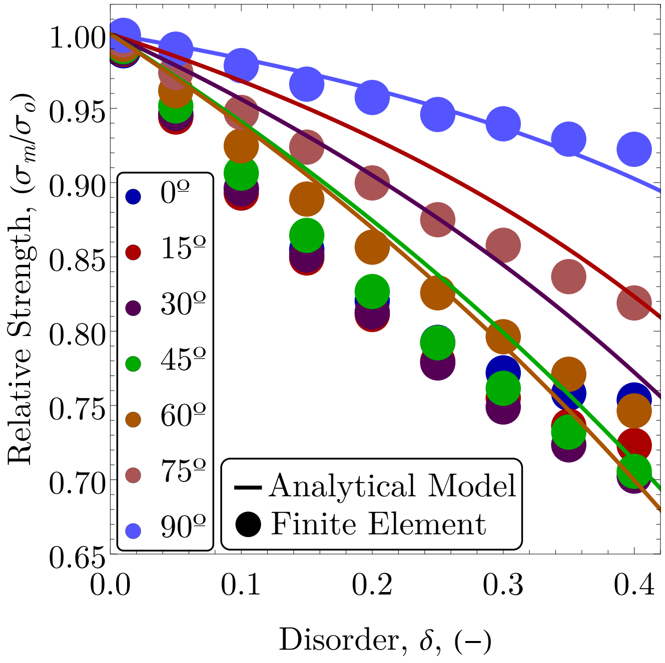

Fig. S4. Finite element results showing relative unit cell strength as a function of disorder for perturbations of varying angles. Predictions from the analytical model are shown as solid lines.

4. Lattices of Varying Unit Cell Size

To verify that the enhancements in toughness through the introduction of disorder were not specific to the case of $L = 5$ mm unit cells, an additional set of simulations were performed of a limited number of ordered and disordered geometries for varying unit cell sizes. The overall dimensions of the double cantilever-beam specimens were preserved, thus, changing unit cell size results in lattices of different numbers of unit cells. As the toughness of a lattice is predicted to primarily scale with density (23-26), the density of the lattices were preserved by keeping the ligament width-to-length ratio fixed at 1/10.

As shown in Fig. S5(A), the toughness of ordered lattices scales linearly with unit cell size, L, as predicted by (25). A linear fit is shown and has an excellent quality of fit ($R^2 = 0.999$). This indicates that the same lattice fracture mechanics framework used for the $L = 5$ mm unit cells and given in (25) should also apply to lattices with unit cells ranging from 3 mm to 7 mm.

Fig. S5(B) shows the lattice toughness, relative to the ordered lattice of the same unit cell size, as a function of disorder. The results for the case of $L = 5$ mm are reproduced from Fig. 4(D), along with results for $L = 3$ mm and $L = 5$ mm. In all cases, the toughness is enhanced relative to the ordered lattice. The maximum enhancement, relative to the ordered lattice, decreases with increasing unit cell size as predicted by eq. (3), since larger unit cells have a smaller total number of ligaments, which places a limit on the amount of distributed damage and the associated toughness. Smaller unit cells are able to achieve large enhancements in toughness even at small levels of disorder; however, as the toughness of the ordered lattice is also reduced for smaller unit cells, this does not generally result in lattices with overall higher toughness than lattices with larger unit cells.

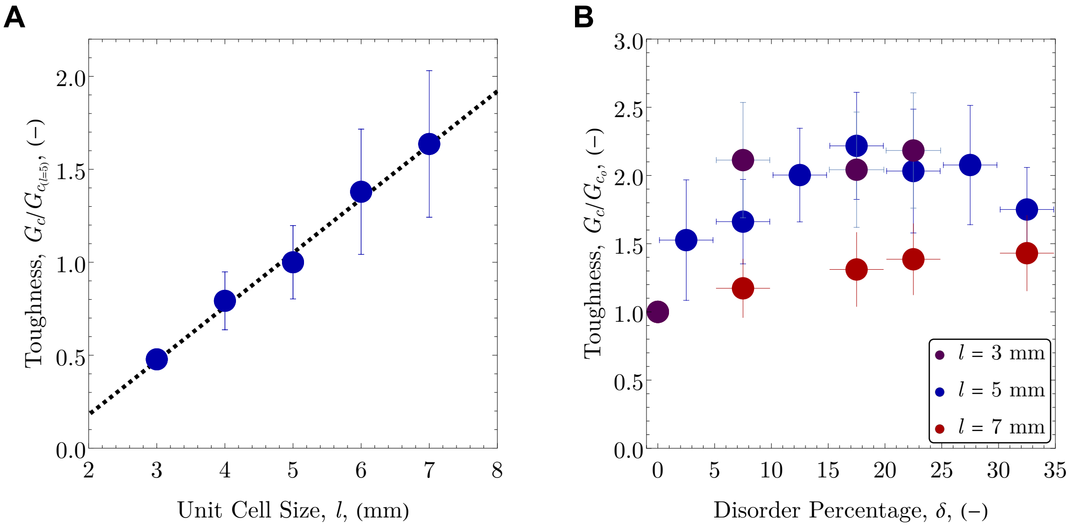

Fig. S5. (a) Finite element-predicted toughnesses of ordered lattices as a function of unit cell size, l, relative to the toughness of an ordered lattice with $l = 5$ mm. A linear fit is also indicated. (b) Finite element-predicted toughnesses, relative to the ordered lattices of varying unit cell sizes, as a function of disorder percentage.

5. Toughness During Crack Propagation

As noted in the Materials and Methods section, toughness results given in Fig. 4(D) were averaged over the total crack growth of each specimen. This ensured the toughness values were not skewed by outlier data points. However, for the average toughness to be an accurate characterization of the fracture resistance of the lattice, the toughness must not vary significantly with crack growth.

Fig. S6 shows the complete simulation and representative experimental toughnesses as a function of crack growth, with neither showing any systematic trends in toughness. Fig.S6 (A) shows the average and standard deviation of the toughness of all simulated geometries, relative to their average toughness, as a function of crack growth. This represents the statistical variation of the over 16,500 simulation data points, and there is no apparent systematic trend in the toughness as a function of crack growth.

Fig. S6(B) shows the toughness as a function of crack growth for a representative specimen of each experimental geometry tested, including the ordered lattice and both seed geometries at varying levels of disorder. While there is variation in the toughness along the crack path due to the non-periodic lattice structures, these variations are, on average, randomly distributed around the average toughness and show a similar level of variation as the finite element simulations.

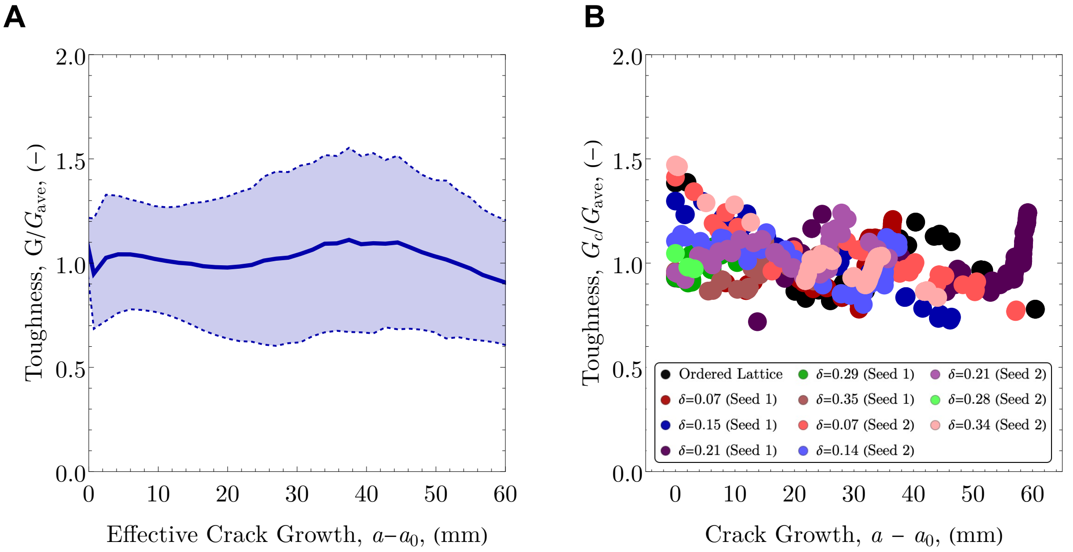

Fig. S6. (a) Mean and std. dev. of the toughnesses of simulated lattices as a function of the effective crack growth, with each individual lattice normalized by its average toughness, G_{ave}; (b) Representative experimental toughnesses of each lattice geometry tested, relative to is average toughness, as a function of the crack growth.

6. Movie Legends

Movie S1. Finite element simulations of the fracture of an ordered lattice with von Mises stresses indicated. Stresses are given for a fixed displacement of 1 mm, and an elastic modulus of 1 GPa. Additional details regarding the simulations are provided in the Materials and Methods section.

Movie S2. Finite element simulations of the fracture of a disordered lattice with von Mises stresses indicated. Stresses are given for a fixed displacement of 1 mm, and an elastic modulus of 1 GPa. Additional details regarding the simulations are provided in the Materials and Methods section.

Movie S3. Photoelastic fracture experiment of an ordered PMMA lattice with ligament failures indicated by red/white points.

Movie S4. Photoelastic fracture experiment of a disordered PMMA lattice with ligament failures indicated by red/white points.
